# Antidepressants are a rational complementary therapy for the treatment of Alzheimer's disease

**DOI:** 10.1186/1750-1326-5-10

**Published:** 2010-03-12

**Authors:** Marwa Aboukhatwa, Laura Dosanjh, Yuan Luo

**Affiliations:** 1 Department of Pharmaceutical Sciences, School of Pharmacy, University of Maryland, 20 N Pine St, Baltimore, MD 21201, USA

## Abstract

There is a high prevalence rate (30-50%) of Alzheimer's disease (AD) and depression comorbidity. Depression can be a risk factor for the development of AD or it can be developed secondary to the neurodegenerative process. There are numerous documented diagnosis and treatment challenges for the patients who suffer comorbidity between these two diseases. Meta analysis studies have provided evidence for the safety and efficacy of antidepressants in treatment of depression in AD patients. Preclinical and clinical studies show the positive role of chronic administration of selective serotonin reuptake inhibitor (SSRI) antidepressants in hindering the progression of the AD and improving patient performance. A number of clinical studies suggest a beneficial role of combinatorial therapies that pair antidepressants with FDA approved AD drugs. Preclinical studies also demonstrate a favorable effect of natural antidepressants for AD patients. Based on the preclinical studies there are a number of plausible antidepressants effects that may modulate the progression of AD. These effects include an increase in neurogenesis, improvement in learning and memory, elevation in the levels of neurotrophic factors and pCREB and a reduction of amyloid peptide burden. Based on this preclinical and clinical evidence, antidepressants represent a rational complimentary strategy for the treatment of AD patients with depression comorbidity.

## 1. Classes of antidepressants

The monoamine hypothesis postulates that depletion in the levels of serotonin, norepinephrine, and/or dopamine in the central nervous system are the pathophysiologic basis of depression. There are five major classes of antidepressants that are categorized according to their mechanism of action on brain amines.

### 1.1 Non selective monoamine reuptake inhibitors (NSRI)

**Figure 1:**
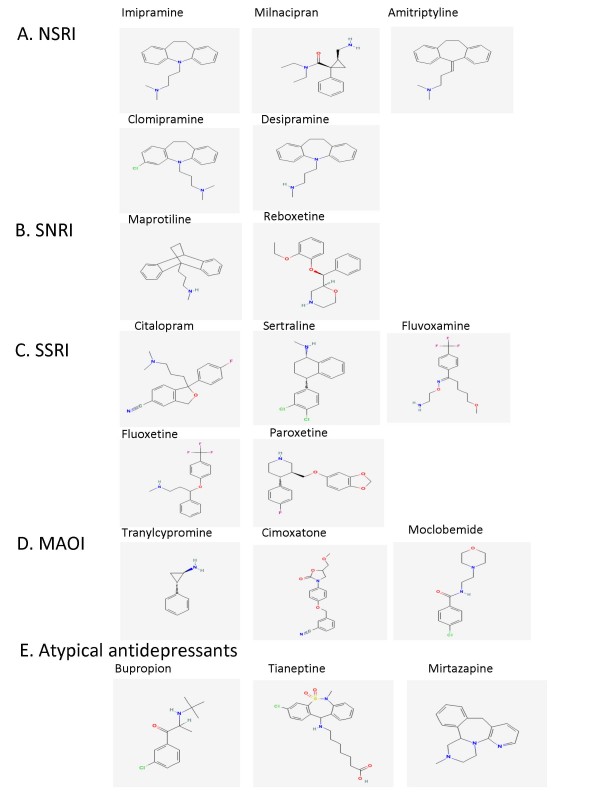
**A: Represents examples of non-selective monoamine reuptake inhibitor (NSRI) antidepressants**. B: Represents examples of selective nor epinephrine reuptake inhibitor (SNRI) antidepressants. C: Represents examples of selective serotonin reuptake inhibitor (SSRI) antidepressants. D: Represents examples of Monoamine oxidase inhibitor (MAOI) antidepressants. E: Represents examples of atypical antidepressants. All the structures are downloaded from PubChem Substance http://pubchem.ncbi.nlm.nih.gov/.

The nonselective monoamine reuptake inhibitor (NSRI) class of antidepressants includes the tricyclic antidepressants (TCA), a group of antidepressants introduced in the 1950s that inhibit the reuptake of both serotonin and noradrenalin. Examples of this class are imipramine, clomipramine, amitriptyline and despiramine (Fig. 1A) [1]. Some reports suggest that dual inhibitors may have superior efficacy and earlier response than selective reuptake inhibitors for a single monoamine [2,3]. In terms of the chemical structure, some TCAs, such as imipramine and amitriptyline, have a tertiary amine structure and are metabolized to secondary amines (Fig. 1A). Other TCAs, such as despiramine and nortriptyline, are secondary amines (Fig. 1A)[4]. In addition to their therapeutic effects; TCAs also have a number of unwanted side effects including antihistaminic, cardiotoxic and anticholinergic effects. These side effects are due to the action of TCAs on adrenergic receptors (α_1_), Na^+^, Ca^2+ ^cardiac channels, histamine (H1) and muscarinic receptors [5-7]. The prescription of TCAs has declined due to these unwanted side effects and the advantage of new antidepressants with a better tolerability profile [4].

There are other non-selective monoamine inhibitors that are structurally different from TCAs but share a similar mechanism of action. Examples of these agents are venlafaxine, duloxetine and milnacipran. Venalafaxine is a derivative of bicyclic phenethylamine and is a more potent inhibitor of serotonin reuptake than norepinephrine reuptake, in addition to low dopamine reuptake inhibition [6,8]. Milnacipran inhibits the reuptake of serotonin and norepinephrine with a similar potency and a negligible effect on dopamine reuptake (Fig. 1A) [9]. Clinical trials of duloxetine in the United States have demonstrated its efficacy in major depressive disorders, particularly those associated with physical pain [10,11].

### 1.2 Selective noradrenaline reuptake inhibitors (SNRI)

The selective norepinephrine reuptake inhibitor (SNRI) class of antidepressants selectively inhibits the reuptake of noradrenaline. Examples of this class are maprotiline and reboxetine (Fig. 1B) [1]. Maprotiline causes side effects similar to those of TCAs including dry mouth, fatigue and weight gain. Reboxetine formulations typically consist of a racemic mixture where the (S) enantiomer is 20 times more potent than the (R) enantiomer [12,13]. The primary unwanted side effects of reboxetine are cardiovascular and urinary effects.

### 1.3 Selective serotonin reuptake inhibitors (SSRI)

**Figure 2:**
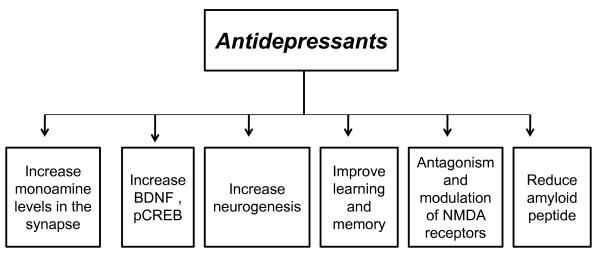
**Summary of different actions of antidepressants that can modulate the pathological features of Alzheimer's disease**.

The selective serotonin reuptake inhibitor (SSRI) class includes antidepressants that selectively inhibit the reuptake of serotonin and subsequently increase the amount of serotonin available to bind to the postsynaptic receptor. SSRIs are the most commonly prescribed class of antidepressants. Examples of this class are citalopram, sertaline, fluvoxamine, fluoxetine and paroxetine (Fig. 1C) [1]. Though these compounds have different pharmacokinetic profiles and chemical structures, they are all metabolized primarily by oxidation prior to excretion [14]. In terms of chemical structure (Fig. 2), fluoxetine has a side chain of propylamine similar to TCAs while citalopram has a dimethyl aminopropyl side chain (Fig. 1C). Paroxetine, sertraline and fluvoxamine are derived from phenylpiperidine, tetrahydronaphthalene and arylketone respectively (Fig. 1C). The major advantage of the introduction of SSRIs in the 1980s was their good safety and tolerability profiles. These favorable profiles are attributed to the low affinity of SSRIs to histamine, muscarinic and α adrenergic receptors. Although SSRIs have a good safety profile, it is important to note probable drug-drug interactions due to an inhibitory effect by some SSRIs on the P450/2D6 cytochrome enzyme [15]. Additionally, reports show that SSRIs have a similar effect as TCAs on K^+^, Ca^2+ ^and Na^+ ^cardiovascular channels, which may contribute to the cardiovascular effects reported in some patients [7,15,16]. Also, sexual dysfunction is a significant side effect that has been reported for SSRIs [17].

### 1.4 Monoamine oxidase inhibitors (MAOI)

The monoamine oxidase inhibitor (MAOI) class of antidepressants inhibits monoamine oxidase (MAO), the enzyme responsible for the metabolism of monoamines. An example of this class is tranylcypromine (Fig. 1D) [1]. Tranylcypromine irreversibly and nonselectively binds to MAO-A and MAO-B. There is a high tendency for hypertensive crisis associated with the use of irreversible and nonselective MAOIs with the concomitant ingestion of tyramine [4]. The development of selective and reversible inhibitors of MAO-A has provided a better safety profile [4]. MAO-A metabolizes the amines that play a major role in depression etiology. Examples of new agents selective for MAO-A are cimoxatone and moclobemide, derived from benzonitrile and benzamide respectively (Fig. 1D). Due to dangerous dietary and drug interactions, the use of MAOIs is generally reserved for patients who don't respond well to other antidepressants or suffer from atypical depression [18,19].

### 1.5 Atypical antidepressants

Atypical antidepressant agents produce an antidepressant effect, but their mechanism of action is not based on the monoamine hypothesis. Examples of this class are bupropion, tianeptine and mirtazapine (Fig. 1E). These compounds have well characterized mechanisms of action, but these mechanisms may not necessarily account for the antidepressant effects [1]. Bupropion inhibits the reuptake of dopamine, tianeptine stimulates the uptake of monoamines, and mirtazapine antagonizes α_2 _adrenergic receptors [1].

There are also many other classes of antidepressants that have been developed recently that are beyond the scope of this review. These drugs have different targets such as the dopaminergic system, serotonin receptors, adrenergic receptors and neuropeptide receptors (for review see [4]).

## 2. Depression and Alzheimer's disease comorbidity

Substantial evidence suggests that depression can be considered both a cause and consequence of a number of neurologic disorders, but the biological link between these disorders has not been determined yet [20]. Depression is considered causative because it is a risk factor for AD [21] particularly if a depressive episode is evident within two years of a dementia diagnosis. In such cases, the depressive episode is considered an early symptom of dementia [22-24]. Depressive symptoms are commonly detected before AD patients manifest cognitive deterioration or are clinically diagnosed [25-27]. Depression instigates a number of complications for AD patients including an increase in mortality, compromise of cognitive function [28] and hindrance in daily living activities [29].

The prevalence rate of depression and AD comorbidity is estimated to be 30-50% [30]. The comorbidity between these two diseases is heterogeneous and is consequently divided into more descriptive subtypes [30]. This categorization takes into consideration the fact that depression can be a risk factor for the development of AD [31] or it can be secondary to the neurodegenerative process [32]. Several pathological events have been discovered that provide a mechanistic link between these two diseases. Comorbidity may be due to depletion of the central superior raphe nucleus [33] or locus coeruleus neurons [34]. Additionally, high levels of glucocorticoids are secreted during depressive episodes that may later have dramatic effects on the hippocampus and lead to dementia symptoms [35].

There are also factors identified that increase the risk of depression development in AD patients. These factors include AD onset at a young age, a family history of mood disorders or depressive symptoms, and female sex [36]. Strong evidence suggests that depressive episodes can be a predictive measure for cognition loss among elderly people who suffer from moderate cognitive impairment [28].

The comorbidity between these diseases poses an impact not only on the patient but also on the caregiver who may suffer higher levels of stress due to the disturbances in the behavior of patients as a result of depression [37]. Caregiver depression is related to patient depression, a consequence that leads to hindrance in the delivery of adequate patient care [38]. It is possible to alleviate the depressive symptoms in both the patient and caregiver groups using certain behavioral interventions that target the patient and involve caregiver participation [39].

A clinical study on the homebound elderly reported that elderly who are non-ApoE4 allele carriers with depression symptoms exhibit lower levels of Aβ 42 and consequently higher plasma ratio of Aβ40/Aβ42 in comparison to non-ApoE4 carriers without depressive symptoms. Because a high Aβ40/Aβ42 ratio is considered to be a risk factor for AD, depression is thought to be a risk factor in the absence of ApoE4 [40]. Another study of geriatric depression reported preliminary findings of high levels of Aβ42 and a high ratio of Aβ42/Aβ40 in patients with late onset depression [41,42]. The exact relationship between declines in cognitive function and plasma levels of amyloid peptide remains to be determined in patients who suffer from AD and depression comorbidity.

### 2.1 Diagnosis challenges

Diagnosis of depression itself is challenging due to the absence of objective diagnostic tests. There is a shortage in the available knowledge of the neuronal circuitry that is involved in depression, and it is unclear where a biopsy should be taken from depressed patients. The heterogeneity of depression adds to the complexity of the diagnosis as well since multiple brain regions are likely to be involved [1].

Diagnosing depression associated with neurological disorders poses further challenges. These diseases frequently have overlapping symptoms and exhibit a similar etiology. Aphasia is an example of an overlapping symptom, which interferes with the patient and physician's ability to communicate regarding the emotional state [43]. Additionally, a reduction in the levels of neurotransmitters such as serotonin and norepinephrine is a similar etiology between AD and depression [44-47]. There is an urgent need for standardized protocols for the diagnosis of depression associated with AD [30]. This need is reflected by an ongoing effort by the National Institute of Mental Health (NIMH) to develop a standard protocol for the diagnosis of depression in AD [48].

### 2.2 Treatment challenges

One of the major treatment challenges is the lack of a clear treatment guide in these patients. The research methodology and the diagnostic criteria heterogeneity confound the clinical results. Another challenge is the strong placebo effect recorded for antidepressant treatment as seen in clinical trials with the TCAs clomipramine [4] and imipramine [49]. The major treatment goal for depression comorbid with other neurological disorders is to relieve the depressive symptoms and improve the coping resources for the neurologic disorder. Research that addresses the depression comorbidity with AD will lead to better treatment outcomes and may also lead to a better understanding of the neuroanatomy of depression [43].

## 3. Preclinical studies on mechanisms of the antidepressants in relation to Alzheimer's disease

### 3.1 Antidepressants stimulate neurogenesis

Recently several groups have demonstrated that neurogenesis exists in the adult brain mainly in two regions. These regions are the subventricular zone (SVZ) and subgranular zone (SGZ) of the dentate gyrus in the hippocampus, an area of the brain that is known to have an important role in learning and memory [50-53]. Reduction in neurogenesis in the SGZ is related to impairment of cognition associated with the aging process and AD, and it may greatly affect the progression of AD [50,54]. Reduction in neurogenesis is implicated in the early symptoms of AD such as impairments in acquiring information and eventually storing it [55]. This is particularly evident in some AD animal models such as mice with the APP and presenilin mutations, which have impairment in dentate gyrus neurogenesis [56-61]. This impairment has led to the introduction of endogenous neuronal precursors as a therapeutic strategy for AD [61-66]. The triple transgenic (3×Tg-AD) AD mouse model that carries mutations in the amyloid precursor protein (APP_*swe*_), tau_*p*301*L *_and presenilin 1PS1_*M*146*V *_exhibits amyloid peptide and tau pathology resembling the human AD brain [67,68]. The triple transgenic (3×Tg-AD) mouse model exhibits an age dependent reduction in adult neurogenesis. At 9 months of age, male 3×Tg-AD mice have approximately a 73% reduction in the generation of new neurons; after 12 months neurogenesis is completely diminished. The reduction in neurogenesis has been correlated to the existence of amyloid peptide plaques and elevation in the number of hippocampal neurons containing amyloid peptide [69]. This study highlights the importance of early intervention to rescue neurogenesis in AD patients, which may then delay the progression of cognitive impairment. There are new strategies to replenish neuronal loss in AD by stimulating endogenous neurogenesis and transplanting neuronal progenitors (NP) [70].

Depression and stress may also decrease neurogenesis and chronic treatment with antidepressants can antagonize this effect and increase neurogenesis in the hippocampus [64,71]. Interestingly, the effects of antidepressants on neurogenesis are evident across different classes including the SNRIs, SSRIs, MAOIs and atypical antidepressants. This neurogenic effect requires chronic administration between 14-21 days, and includes an increase in the proliferation rate and new neuron survival [72]. The underlying mechanisms that mediate the neurogenic effects of antidepressants have not been identified, but there is strong evidence that neurotrophic factors such as fibroblast growth factor-2, insulin-like growth factor-1 (IGF-1) and brain derived neurotrophic factor (BDNF) are important for this effect [4]. It has been reported that the increase in the new neuron survival rate but not the proliferation rate is dependent on BDNF [73]. Antidepressant activation of the CREB pathway has also been implicated as an important component underlying the neurogenic effect [74]. Fluoxetine (SSRI) treatment for as short as 5 days can increase synaptic density in the hippocampus as determined by electron microscope [75]. In contrast, amitriptyline (TCA) treatment does not increase the number of synapses but reduces declines in synaptic density as a result of olfactory bulbectomy, a well established animal model for depression [76]. Chronic tiapentine (atypical antidepressant) treatment prevents reduction of dendrite length as a result of chronic stress [77]. Behavioral studies imply that the neurogenic effect of antidepressants is required to mediate antidepressant action. In a study by Santarelli et al., cell proliferation was inhibited by irradiation and subsequently blocked antidepressant action in chronic unpredictable stress and novelty suppressed feeding [78]. Chronic unpredictable stress and novelty suppressed feeding are depression animal models that require long term treatment with antidepressants to produce antidepressant action and are particularly relevant when the role of the neurogenesis is investigated [79]. In contrast, the tail suspension and forced swim tests require acute administration of antidepressants to produce antidepressant action. Chronic administration of antidepressants corresponds to the time frame that is required for the maturation and differentiation of new neurons to functional ones [80].

It is important to mention that acute versus chronic administration is considered a variability factor in the forced swim test [81]. The forced swim testis utilized to screen for acute antidepressant effects, although a chronic time course is required for the clinical effects [81]. A study addressing the effect of fluoxetine on the forced swim test after different dose intervals demonstrated that chronic administration can enhance the effects seen at acute or subchronic dosing [82]. This study calls attention to the weak face validity of the forced swim test [81].

Based on these animal studies where a reduction in neurogenesis was demonstrated to lead to cognitive impairment and the ability of antidepressants to stimulate adult neurogenesis, antidepressant treatment may provide AD patients with an advantage. Concurrent antidepressant treatment may increase the proliferation and survival of new neurons, particularly if the treatment is started early when depressive symptoms appear as a risk factor.

### 3.2 Antidepressants stimulate learning and memory

After 65 years of age, the elderly must cope with alterations in memory as a part of the normal aging process. This is evident in recognition memory changes [83] and impairment of spatial memory [84,85]. Also, hippocampal dysfunction may underlie alterations in memory during the aging process, and has been consistently observed across different species [83]. Given that age is an important risk factor for AD where up to 40% of elderly people over 65 years suffer from AD [86], drugs that stimulate learning and memory carry important benefits to AD patients. There are major changes in the hippocampus associated with the aging process such as electrophysiological silence in synapses as a result of reduction in the post synaptic density, difficulty in encoding and retaining information as evident by reduction in long term potentiation (LTP) and elevation in long term depression (LTD) and synaptic contact loss [87]. The main endophenotype of major depression is impairment in cognition [88]. This is clinically evident by difficulty in concentration and attention due to abnormalities and neuropathological changes in dorsolateral prefrontal cortex that is critical to these capacities [89-91]. Interestingly, preclinical studies in animals report that chronic treatment with antidepressants increase LTP and field potential baseline in dentate gyrus in a similar way to chronic electromagnetic stimulation [92,93]. It is speculated that the increase in newborn granule cell number in the dentate gyrus underlies the potential neuroplastic effect [94,95]. On the other hand, earlier reports showed that tricyclic antidepressants reduce LTP in CA1 pyramidal cells [96,97]. The reduction of LTP can be attributed to anticholenergic effects of the TCA that counteract their effects on neuroplasticity [80]. As a proof of concept, chronic treatment with atypical antidepressant (tiapentine) or SSRIs that have less anticholinergic properties in comparison to TCAs increase LTP and prevent stress induced reduction in LTP [98,99]. Another report confirms the beneficial effects of chronic fluoxetine and tiapentine treatment in preventing stress induced reduction of LTP in hippocampus-prefrontal cortex circuitry [100]. Based on these studies, it is evident that chronic administration of SSRIs increases cellular plasticity in dentate gyrus and CA1 pyramidal cells and prevents the harmful effects of stress in hippocampal neurons. Additionally, the anticholinergic properties of TCAs may counteract their neuroplastic effects.

There are conflicting reports on how SSRI treatment affects performance in the Morris water maze, a typical model for spatial learning and memory. Reports demonstrated an improvement in Morris water maze performance after chronic treatment with venlafaxine or fluoxetine [101-103]. Another study reports that fluoxetine does not affect performance in the Morris water maze [93]. Chronic treatment with the atypical antidepressant tiapentine does not affect performance in the Morris water maze [102] but improves performance in the radial maze discrimination task [104]. Chronic treatment with the TCA amitriptyline blocked age induced deterioration of learning and memory [105]. In contrast to SSRIs and amytriptyline, imipramine does not affect performance in the Morris water maze [101] and even worsens spatial working memory in the radial arm maze test [106]. The fact that TCAs impair cognitive function has also been reported in some clinical trials [49,107]. These preclinical studies raise awareness about selection of the proper antidepressant for AD patients. Based on the reports that have shown some antidepressants can cause memory impairment, close attention should be paid to antidepressants prescribed to AD patient [108].

### 3.3 Antidepressants and N-methyl-D-aspartate (NMDA) receptors

Mounting evidence supports the hypothesis that inadequate stimulation of NMDA receptors is a pathophysiological component of both depression and AD. The NMDA receptor represents an interesting treatment target due to the comorbidity between these two diseases [109-112]. Under normal physiological conditions, the synaptic activity of NMDA receptor modulates APP processing towards a direction that favors non-amyloidogenic α-secretase processing of amyloid precursor protein [113]. APP processing by α-secretase is reduced as a result of chronic NMDA receptor stimulation and leads to an increase in amyloid peptide production in the cortical neurons that resembles the pathophysiological conditions of AD [114]. Stress induced hippocampal neuronal atrophy and reduction in neurogenesis can be blocked by NMDA receptor antagonist treatment [115].

A number of studies report that chronic antidepressant treatment can modulate the expression of specific NMDA receptors subunits and ultimately NMDA receptor function [116-120]. NMDA receptor function is reduced after treatment with antidepressants [121-123]. Tricyclic antidepressants inhibit the NMDA receptor directly [124,125]. Milnacipran is a serotonin and norepinephrine reuptake inhibitor that antagonizes the NMDA receptor noncompetitively [126]. The SSRI fluoxetine inhibits the NMDA receptor directly [127].

Additionally, NMDA receptor antagonists such as memantine, 2-amino-7-phosphoheptanoic acid (AP-7), eliprodil, 1-aminocyclopropancarboxylic acid (ACPC), MK-801 and fenprodil have antidepressant-like effects [128-130]. Memantine and MK-801 are noncompetitive NMDA receptor antagonists, AP-7 is a competitive NMDA receptor antagonist, ACPC is partial agonist on the glycine site, and eliprodil and fenprodil work on the polyamine binding site of the NMDA receptor [128-130]. Interestingly, a case report for the antiviral agent amantadine which has NMDA receptor antagonistic activity provides clinical evidence for its efficacy in depression [131]. Ketamine is another NMDA receptor antagonist, which has antidepressant effects after a single dose administration in depressed patients [132]. Ketamine also exhibits antidepressant and anxiolytic effects in animal models of depression [133].

Antidepressant treatment can serve a dual role in patients who suffer from AD and depression. It can treat the depressive symptoms in addition to targeting NMDA receptor activity in AD patients. Of note is the fact that inhibition of NMDA activity is evident in SSRI agents such as fluoxetine, which have a better tolerability profile in comparison to TCAs.

### 3.4 Antidepressants, serotonin, BDNF and pCREB

Serotonin signaling pathways are implicated in the pathology of AD since the death of the neurons and the dysfunction of the synapse can be a result of reduction in the activation of serotonin coupled signaling pathways [134]. Amyloid peptide deposition, a major pathological feature of AD, interferes with the phosphorylation of cAMP-response element-binding protein (CREB) [135]. Intracellular amyloid peptide load affects this signaling pathway differently. Moderate elevation in levels of intracellular amyloid peptide load leads to over expression in CREB responsive genes such as BDNF, presenilin 1 and presenilin 2. High levels of intracellular amyloid peptide lead to persistent CREB hyperphosphorylation and block its translocation to the nucleus resulting in inhibition of cyclic AMP-response (CRE) directed gene expression [136]. The authors speculate that inhibition of CREB translocation causes early synaptic dysfunction prior to the extracellular accumulation of amyloid peptide [136].

Chronic treatment with antidepressants increases the synaptic concentrations of noradrenaline and/or serotonin. These increased levels then lead to activation of G-protein coupled receptors, stimulation of adenyl cyclase, and eventually upregulation of the cAMP cascade. This cascade results in increases of CREB and BDNF expression and increases in the levels of cAMP-dependent protein kinase (PKA) [4,115,137-139]. Serotonin enhancement of synaptic plasticity is mediated by activation of CREB and increases in BDNF levels [140].

Given the high prevalence rate of comorbidity between depression and AD, it is important to screen AD animal models for depressive symptoms. R406 W transgenic mice are an AD animal model with tau hyperphosphorylation, deposition of neurofibrillary tangles in forebrain and impairment in associative memory [141,142]. Interestingly, R406 W transgenic mice have been evaluated in the forced swim test and have been demonstrated to exhibit a longer immobility time than non-transgenic mice [141]. Fluvoxamine not desipramine treatment of R406 W transgenic mice restores immobility time in the forced swim test to wild type levels. This study implies that R406 W transgenic mice demonstrate depressive behaviors and provide evidence for the involvement of serotonin in these depressive symptoms. Indeed, these mice exhibited low levels of 5-hydroxyindoleaceticacid (5-HIAA) and serotonin, and fluoxamine treatment restores serotonin levels comparably to control group. This study raises speculations that the R406W mutation affects serotonergic neurons [141]. Postmortem AD brains show reductions in the levels of serotonin and its metabolites [44,143], which highlight the advantage of prescribing SSRIs to AD patients versus other antidepressants.

There is an association between reduced levels of neurotrophic factors and depressive symptoms, and mounting evidence supports the hypothesis that part of antidepressant action involves increasing levels of neurotrophic factors to compensate for their reduced levels in depressed patients [144,145]. There is a family of structurally related trophic factors that includes BDNF, neurotrophin-3, neurotrophin-4 and nerve growth factor (NGF). Generally, the production of BDNF mRNA results from the stimulation of 5-HT receptor and β-adrenoceptor coupled signaling pathways. The growth and function of serotonergic neurons are greatly increased by BDNF [145,146]. BDNF also reduces mRNA and protein levels of NMDA receptor subunits and reduces NMDA stimulated Ca^2+ ^increase [147]. BDNF and NGF specifically have important effects on hippocampal neurons that are involved in the pathogenesis and clinical features of AD [66,148,149]. It has been reported that the amyloidogenic pathway is activated as a result of NGF deprivation [150] and that BDNF or NGF signaling interruption leads to cell death and accumulation of Aβ aggregates intracellularly and extracellularly [151]. A recent study demonstrated that BDNF gene delivery significantly restored learning and memory, reversed synaptic loss, partially normalized inappropriate gene expression and improved cell signaling in transgenic mice even after disease onset [152]. Neurotrophic factors have now entered clinical trials as both a preventative measure and as a treatment to reduce neuronal loss and stimulate neurogenesis [153,154]. These studies demonstrate that BDNF is likely a key player in mediating the beneficial effects of antidepressants in AD patients.

### 3.5 Antidepressants and amyloid peptide

The effect of antidepressants on amyloid peptide has particular importance. The high prevalence rate of comorbidity between depression and AD warrants the investigation of the possible dual role for antidepressants in modulating these two diseases. Additionally, antidepressants activate similar signaling pathways as the ones activated by dietary restriction and environmental enrichment, both of which have been demonstrated to reduce amyloid peptide burden in transgenic mice [134,155,156].

Chronic treatment with paroxetine for 5 months in 3×TgAD mice significantly reduces the levels of amyloid peptide 1-40 in the hippocampus and cerebral cortex [157]. Tau immunoreactivity is also significantly reduced in the hippocampus and amygdala in paroxetine treated mice [157]. Although the underlying mechanism for the action of paroxetine in reducing amyloid peptide burden and tau pathology is undetermined, there is speculation that the effect is due to enhancement of serotonin signaling and elevation of BDNF expression levels [134,158]. To investigate whether the effect of antidepressants on amyloid peptide is limited to the SSRI class, we examined the effect of increasing concentrations of antidepressants on Aβ expressing N2a neuroblastoma cells by Western blotting. The tested antidepressants include the SSRIs fluoxetine and paroxetine, the selective noradrenaline reuptake inhibitor maprotiline and the nonselective monoamine reuptake inhibitor imipramine. Interestingly, fluoxetine and paroxetine at 10 μM significantly decrease Aβ oligomers, but do not affect the levels of extracellular amyloid peptide (unpublished data). Based on these results, fluoxetine and paroxetine are likely to be beneficial to AD patients due to their role in modulating Aβ metabolism. This effect may also explain some of the beneficial effects of SSRIs in AD patients. In a screening assay for small molecules that can interact with Aβ fibrils, fluoxetine does not show potential to interact with Aβ fibrils directly [159].

Targeting amyloid precursor protein (APP) gene expression is a major anti-amyloid strategy in the treatment of AD. Desferrioxamine and phenserine target the 5' untranslated part of APP and ultimately inhibit APP translation [160]. Interestingly, paroxetine was one of the APP 5'UTR lead directed compounds based on a screening study from a 1,200 compound library [161]. Paroxetine treatment for 48 hours in B3 lens epithelial cells reduces the levels of Aβ secreted into the medium [161,162]. B3 lens epithelial cells were specifically used in this study due to high baseline levels of amyloid peptide [163]. TgCRND8 mice treated with paroxetine for three months had reduced levels of Aβ (1-40) and APP levels in brain homogenate. TgCRND8 mice were selected for this study because the APP gene open reading frame is over expressed in these mice, providing a proof of concept for the APP 5'UTR targeting strategy [163,164].

Another *in vitro *study addressed the effect of TCAs and SSRIs on APP processing in rat primary basal forebrain cultures [165]. Imipramine at 100 μM significantly reduced intracellular levels of APP after two hours of treatment. Imipramine and citalopram significantly increased the levels of secreted APP in the medium of the treated primary cultures [165]. Interestingly, serotonin and muscarinic agonists also increase APP secretion [166-168]. It is anticipated that the increase in APP secretion is accompanied by a decrease in intracellular APP levels. Presumably, the secreted APP will not be available for processing by β and γ secretases [165]. Whether the effect of antidepressants on APP processing and amyloid peptide are a class effect or whether these effects relate to pharmacological mechanisms individual antidepressant agents has not been determined.

### 3.6. Natural antidepressants and AD (Ginkgo, St. John's wort, flavonoids, and curcumin)

St. John's wort (*Hypericum perforatum*) extract (HPE) is well known for its antidepressant effects [169-171]. Hyperforin is considered to be the major active constituent that contributes to the neuroprotective effect of HPE [172]. The antidepressant action of hyperforin is primarily attributed to monoamine reuptake inhibition [173]. Other components in HPE have also been identified to have an important contribution to the antidepressant effect of HPE such as flavonoids [174], pseudohypericin and hypericin [175].

HPE extract has been demonstrated to exhibit neuroprotective properties by preventing the toxic effect of amyloid peptide (25-35) in the hippocampal neurons of the rat. HPE reduced lipid peroxidation, cell death and dendritic lesions [176]. In another study, pretreatment of a microglial cell line with HPE showed a dose dependent reduction in amyloid peptide induced cell death [177]. To study the effect of individual components of HPE on cell viability, individual constituents of the HPE mixture were incubated with the microglial cell line. Some flavonols such as (-)-epicatechin and (+)-catechin increased the viability of the cells but other flavonols and glycosides such as quercitrin, quercetin, hyperosid and rutin had no effect [177]. The antioxidant properties of the flavonoids resulted in reduced reactive oxygen species (ROS) production induced by amyloid peptide in the microglia [177]. Hyperforin in particular has been demonstrated to enhance memory in rodents [178]. Another study reported that hyperforin improved spatial memory by reduction of reactive astrocytes, activation of microglia and promotion of amyloid peptide deposit fragmentation [179]. Hyperforin also protects cells against the neurotoxic effect of amyloid peptide oligomers and fibrils and reduces the production of ROS [179]. An *in vitro *study demonstrated that hyperforin promotes the dissociation of amyloid peptide deposits dose and time dependently and converts the fibrils to protofibrils [179]. These studies provide evidence for the role of hyperforin in improving the memory by reduction of neurotoxic amyloid peptides.

*Ginkgo biloba *leaves are a common herbal remedy in traditional Chinese medicine. Extract of *Ginkgo biloba *leaves (EGB) demonstrated antidepressant action in forced swim test and tail suspension test [180]. The roles of individual constituents in EGB that relate to the antidepressant activity have not been determined. It is likely that terpenoids which represent 6.5% of EGB [181] play a role in antidepressant action based on the reported action in the central nervous system [63,182]. Another study demonstrated antidepressant activity of *Ginkgo biloba *lipophylic extract in learned helplessness and behavioral despair animal models[183]. It 6-alkylsalicylates have also been implicated as active constituents related to the antidepressant activity of the *Ginkgo biloba *lipophylic extract [183].

*Ginkgo biloba *leaves exhibits a number of beneficial effects for AD patients such as cognition and mood improvements and resolution of mild to moderate dementia symptoms [184-189]. Although a recent Ginkgo trial failed to demonstrate prevention of memory impairment, the authors discuss the possibility that the extract was given too late to see a preventive effect [190]. In preclinical studies, Ginkgo biloba extract (EGB 761) blocked the production of amyloid beta peptide and amyloid precursor protein in aged rodents [191]. EGB 761 also inhibits the aggregation of amyloid peptide and apoptosis by blocking the activation of caspase-3 in a neuroblastoma cell line [192]. EGB 761 has also been demonstrated to inhibit amyloid peptide induced hippocampal cell death [193] and increase the levels of phosphorylation of CREB that are reduced as a result of conditioned medium treatment to wild type neuroblastoma cells [194]. A study from our laboratory also demonstrated the neurogenic potential of EGB761 in an AD mouse model where it induced an increase in cell proliferation and neuronal precursor cells numbers in hippocampus [195].

Flavonoids are class of compounds that are derived from different plants such as tea, *Ginkgo biloba *and citrus [196]. Accumulating evidence supports the antidepressant activity of flavonoids in depression animal models [197-199]. Given the fact that depression and AD share common pathophysiological abnormalities of CREB- BDNF signaling pathway, citrus and green tea flavonoids may increase the phosphorylation of CREB and improve the memory [200,201]. Recently we have reported that Ginkgo flavonols activate signaling pathways, which are heavily implicated in depression including the BDNF/pCREB pathway. Additionally, Ginkgo flavonols also reduced amyloid peptide burden in double transgenic (TgAPPswe/PSe9) mouse hippocampal neurons [202].

## 4. Clinical studies of antidepressants on cognitive function in AD patients

### 4.1 Antidepressant clinical studies

In general SSRIs have a better tolerability and safety profile when compared to TCAs. Citalopram is an SSRI that has been shown to significantly improve the score of depressed patients in the Hamilton Rating Score (HAM-D), the Clinical Global Impression Scale, and the Montgomery Asberg Depression Scale (MADRS) [203]. Citalopram also significantly improves emotional and cognitive function in a subgroup of patients who suffer from dementia based on the Gottfries-Brane-Steen Dementia Rating Scale [203].

The SSRI sertraline was tested in an 8-week trial in 31 female patients diagnosed with late stage AD to determine its efficacy. Using objective rating scales, including the Cornell Scale for Depression in Dementia and others, sertraline and placebo improve ratings similarly but sertraline treatment showed a better improvement in "knit brow" facial behavior [204]. "Knit brow" is facial behavior where the brows are somewhat lowered and pulled together. It is a robust index of dysphoria in advanced stage dementia [204]. Another clinical study with sertraline treatment that lasted 12 weeks involving 22 patients who suffer from major depression and AD showed that sertaline reduced depressive symptoms significantly in comparison to placebo. Interestingly, sertraline treated patients do not show any significant change in daily living activities according to the Psychogeriatric Dependency Rating Scale in comparison to the placebo group where there was a significant decline in daily activities at weeks 9 and 12 [205].

A meta analysis study for the safety and efficacy of antidepressants in treatment of depression in AD found that antidepressants are efficacious in treatment of depression in AD patients and have a similar dropout rate as placebo [206].

### 4.2 Combination studies

There are number of reasons for the initiation of combinatorial studies that include antidepressants and other FDA approved drugs for the treatment of AD. First, impairment in the cholinergic system does not fully account for age-associated cognitive declines [65,207]. AchEIs also improve behavioral and non-cognitive aspects [208,209]. Secondly, there is evidence of oxidative stress, neuroinflammation in the postmortem brain of AD patients. It has been suggested that other neurotransmitter systems are involved such as the glutamatergic and serotonergic systems [210]. Abnormalities in monoaminergic systems have been reported in AD patients [211,212] and AD patients have lower levels of the neurotransmitter serotonin (5-HT) and its metabolites [213,214]. Accumulating evidence emphasizes the positive role that serotonin plays in cognitive function [215]. Improvements in both immediate and delayed verbal memory after treatment with SSRIs indicate an enhancement in hippocampal function [62,216]. Also, the efficacy of memantine for treatment of moderate-to-server dementia of AD patient supports the notion that cholinergic impairment do not fully account for age-associated cognitive decline. These factors provide a rational argument for the potential beneficial effects for combinatorial studies between antidepressants and other FDA approved drugs for the treatment of AD.

To test if the addition of an SSRI (fluoxetine) to an acetylcholinesterase inhibitor (rivastigmine) treatment regimen could benefit AD patients, a double blind placebo controlled study was conducted for 12 weeks in patients with mild to moderate dementia between the ages of 55-85 years. This study included 122 patients divided into three treatment groups: placebo, rivastigmine only and rivastigmine plus fluoxetine. The results of this report showed that there were improvements in cognition and memory in rivastigmine treated and rivastigmine plus fluoxetine treated groups but without a significant difference between these two groups. Interestingly, the rivastigmine plus fluoxetine treatment group had better performance in daily life activities and overall function which highlights the benefits that may be obtained by adding a serotonin regimen to FDA approved drugs for AD patients [216]. Another study also reported the beneficial outcome of combining sertaline (SSRI) with donepzil treatment especially for AD patients with moderate to severe dementia [217].

The interplay between the cholinergic and the serotonergic systems has an important relevance to AD as suggested by a number of studies. There is neurochemical and neuroanatomical evidence for the role of cholinergic system in modulating the serotonergic one and the potential synergism between them in improving memory function [218-223]. A recent study reports that the acute administration of citalopram reduced glucose metabolism in the brain while the concomitant administration of acetylcholinesterase inhibitor and nicotinic receptor modulator (galantamine) and citalopram have increased glucose metabolism. These data suggest a beneficial interplay between the cholinergic and serotonergic systems for AD patients [222].

These clinical studies in addition to preclinical evidence support the positive role of chronic administration of SSRIs in hindering the disease progression and improving AD patient clinical outcomes [157,224].

## Conclusion

In this review, we highlighted the high prevalence rate of comorbidity between AD and depression and summarize different potential targets for antidepressant drugs that may relate to the AD pathology (see Table 1). Neurodegeneration associated with AD involves different neurotransmitter systems such as the glutamenergic, serotonergic, peptidergic and cholinergic systems [225]. There are myriad of reasons to consider antidepressants as an adjunct treatment to AD patients, several of which were discussed in this review. Additionally, we summarized some of the clinical evidence that demonstrated the beneficial effects of SSRIs in AD patients either alone or in combination with other FDA approved acetylcholine esterase inhibitors. The preclinical studies present potential targets that may underlie antidepressants mechanisms of action in AD pathology including neurogenic effects, stimulation of learning and memory, antagonism of NMDA receptors, reduction of amyloid peptide burden and upregulation of neurotrophic factors.

**Table 1:** Summary of potential targets of antidepressant drugs in relate to AD pathology

*Antidepressants*	*Neurogenesis*	*Aβ*	*Learning & memory*	*NMDA Receptors*
Fluoxetine (SSRI)	Increase synaptic density in hippocampus[75]	Does not interact with Aβ fibrils [159].	Protects hippocampal LTP [100]. Performance improvement in Morris water maze after chronic treatment [102].	Inhibit NMDA receptor directly [127].

Amitriptyline (NSRI)	Does not increase synapse number but reduce decline in synaptic density [76].		Blocks age --induced deterioration of learning and memory [105].	

Tiapentine (atypical)	Prevents the reduction of dendrites length as a result of chronic stress [77].		Protects hippocampal LTP [99,100]. No effects on animal performance in Morris water maze[102] but improve animal performance is radial maze discrimination task [104].	

TCA			Reduce LTP in CA1 pyramidal cells [96,97].	Inhibit NMDA receptor directly [124,125].

Venlafaxine (SNRI)			Performance improvement in Morris water maze after chronic treatment [101,103].	

Imipramine (NSRI)		Increase secreted APP, reduces intracellular APP in culture [165].	No effect on animal performance in Morris water maze [101] and even worsen spatial working memory in radial arm maze test [106].	Changes in binding to NMDAR [118,120]and expression of NMDAR in brain [116]

Citalopram (SSRI)		Increase the levels of secreted APP in the medium of the treated neurons [165].		Adaptation of NMDAR complex [117]. Changes in expression of NMDAR [116].

Clomipramine (NSRI)				Chronic administration changes the regulation of NMDA receptor control on the release of dopamine [119].

Milnacipran (NSRI)				Antagonize NMDA receptor uncompetitively [126].

Paroxetine (SSRI)		Reduces levels of Aβ and tau in Tg mice and cells [157,161-164]		

## List of Abbreviations

NSRI: non selective monoamine reuptake inhibitor; SNRI: selective norepinerphrine reuptake inhibitor; SSRI: selective serotonin reuptake inhibitor; AD: Alzheimer's disease; TCA: Tricyclic antidepressants; MAO: Monoamine oxidase; MAOI: M MAOI: Monoamine oxidase inhibitor; HAM-D: Hamilton Rating Score; MADS: Montgomery Asberg Depression Scale; NP: Neuronal progenitors; IGF-1: Insulin-like growth factor-1; BDNF: Brain derived neurotrophic factor; LTD: Long term depression; LTP: Long term potentiation; NMDA: N-methyl-D-aspartate; AP-7:2-amino-7-phosphoheptanoic acid; ACPC: 1-aminocyclopropancarboxylic acid; CREB: cAMP-response element-binding protein; 5-HIAA: 5-hydroxyindoleaceticacid; NGF: Nerve growth factor; APP: Amyloid precursor protein (APP); *HPE: Hypericum perforatum *extract; EGB: Extract of *Ginkgo biloba *leaves

## Competing interests

The authors declare that they have no competing interests.

## Authors' contributions

MA searched literature, wrote the draft and revised the manuscript; LD added additional information, edited and revised the manuscript; YL provided editing and financial support. All authors read and approved the final manuscript.
